# Vacuum-Induced Degradation of 2D Perovskites

**DOI:** 10.3389/fchem.2020.00066

**Published:** 2020-02-13

**Authors:** Yvonne J. Hofstetter, Inés García-Benito, Fabian Paulus, Simonetta Orlandi, Giulia Grancini, Yana Vaynzof

**Affiliations:** ^1^Kirchhoff Institute for Physics and the Centre for Advanced Materials, Heidelberg University, Heidelberg, Germany; ^2^Integrated Centre for Applied Physics and Photonic Materials and Centre for Advancing Electronics Dresden (CFAED), Technical University of Dresden, Dresden, Germany; ^3^Group for Molecular Engineering of Functional Materials, Institute of Chemical Sciences and Engineering, Sion, Switzerland; ^4^CNR - Istituto di Scienze e Tecnologie Chimiche “G. Natta” (CNR-SCITEC), Milan, Italy; ^5^Department of Chemistry, University of Pavia, Pavia, Italy

**Keywords:** 2D perovskites, stability, vacuum, cations, X-ray photoemission spectroscopy

## Abstract

Two-dimensional (2D) hybrid organic-inorganic perovskites have recently attracted the attention of the scientific community due to their exciting optical and electronic properties as well as enhanced stability upon exposure to environmental factors. In this work, we investigate 2D perovskite layers with a range of organic cations and report on the Achilles heel of these materials—their significant degradation upon exposure to vacuum. We demonstrate that vacuum exposure induces the formation of a metallic lead species, accompanied by a loss of the organic cation from the perovskite. We investigate the dynamics of this reaction, as well as the influence of other factors, such as X-ray irradiation. Furthermore, we characterize the effect of degradation on the microstructure of the 2D layers. Our study highlights that despite earlier reports, 2D perovskites may exhibit instabilities, the chemistry of which should be identified and investigated in order to develop suitable mitigation strategies.

## Introduction

**Figure 1:**
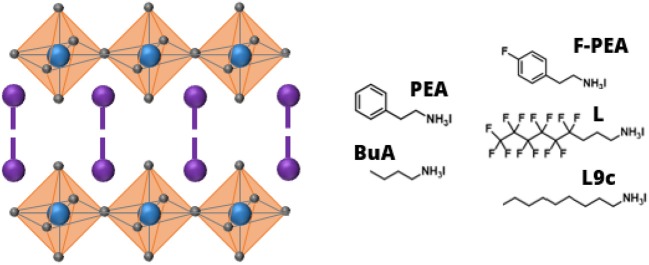
Schematic structure of the 2D perovskite with an empirical formula of (cation)_2_PbI_4_ **(left)** and the molecular structures of the organic cations investigated in this work **(right)**.

Lead halide perovskites are a fascinating class of materials with superior optoelectronic properties (Stoumpos et al., 2013; Stranks et al., 2013; Xing et al., 2013; Green et al., 2014; Saba et al., 2014; DeQuilettes et al., 2015; Dong et al., 2015). Their properties can be controlled by tuning their composition (Roldán-Carmona et al., 2015; Saliba et al., 2016a,b; Yang et al., 2017; Fassl et al., 2018), defect density (Buin et al., 2014; Ball and Petrozza, 2016; Fassl et al., 2019b), microstructure (Grancini et al., 2015; Sun et al., 2018; Xu et al., 2018; Fassl et al., 2019a), and dimensionality (Quan et al., 2016; Gao et al., 2018) among other examples. In particular, low dimensional 2D perovskites have recently attracted the attention of the scientific community and found application in a range of optoelectronic devices (Cao et al., 2015; Byun et al., 2016; Chen and Shi, 2017; Cohen et al., 2017; Etgar, 2018; Grancini and Nazeeruddin, 2019). In 2D perovskites, the inorganic perovskite sheet is isolated by long organic cations, creating a layered structure as shown in Figure 1. The structure is well-defined only for a monolayer of the inorganic sheet (*n* = 1), but for higher values of n, a mixture is formed containing also lower n perovskites, so such structures are termed quasi-2D perovskites. Unlike 3D perovskites that severely degrade upon exposure to environmental conditions such as oxygen (Pearson et al., 2016; Aristidou et al., 2017; Pont et al., 2017; Sun et al., 2017), and water (Mosconi et al., 2015; Ma et al., 2017; Wang Q. et al., 2017) 2D and quasi-2D perovskites exhibit a far enhanced stability and robustness to water (Smith et al., 2014; Tsai et al., 2016; Chen et al., 2017; García-Benito et al., 2018). This has led to their incorporation in photovoltaic devices either as a capping layer of a 3D perovskite layer (Cao et al., 2015; Ma et al., 2016; Grancini et al., 2017; Cho et al., 2018) or mixed with a 3D perovskite in a 2D-3D stacking- or heterostructure (Wang Z. et al., 2017; Lin et al., 2018), resulting in a significant increase in device stability.

Apart from poor environmental stability, 3D perovskites have also been shown to similarly degrade under vacuum conditions, however more slowly than in air (Alberti et al., 2015). In particular, it has been shown that methylammonium iodide sublimes in vacuum, leading to the formation of lead iodide (Deretzis et al., 2015; Juarez-Perez et al., 2018; Alberti et al., 2019). Exposure to vacuum has also been shown to lead to the formation of metallic lead in 3D perovskites (Gunasekaran et al., 2018) and to the reduction of photoluminescence (Fang et al., 2016; Tang et al., 2016). To date, the effect of vacuum on 2D perovskites remained unexplored.

In this work, we investigate the effect of vacuum exposure on 2D perovskites (*n* = 1) with five different organic cations, the chemical structure of which is shown in Figure 1. We follow the evolution of the composition of the 2D perovskite samples and quantify the dynamics of their degradation. We also characterize the microstructural changes in the films. Our results demonstrate that 2D perovskites are sensitive to vacuum with the choice of cation strongly affecting the stability of the 2D perovskite.

## Results and Discussion

### Effect of Exposure to Vacuum on the Composition of 2D Perovskites

**Figure 2:**
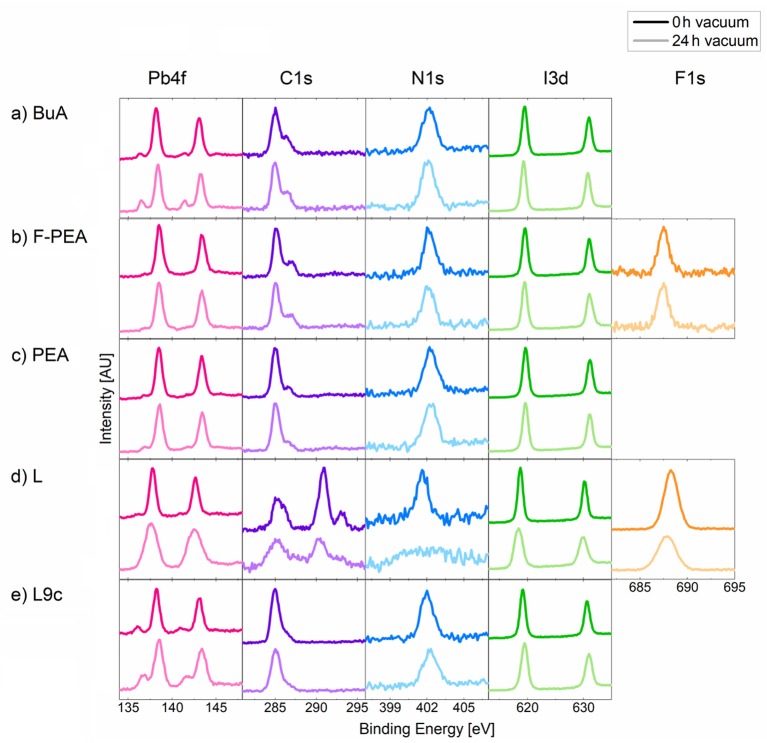
XPS spectra of the 2D perovskites with organic cations **(a) BuA**, **(b) F-PEA**, **(c) PEA**, **(d) L** and **(e) L9c** at 0 and 24 h in vacuum. The XPS peaks were shifted so that the C-C peak is at a binding energy of 285 eV.

To investigate whether exposure to vacuum has an effect on the composition of 2D perovskite layers, surface-sensitive X-ray photoemission spectroscopy (XPS) measurements were performed on pristine samples and samples that were exposed to vacuum for 24 h. It is noteworthy, that since XPS is performed under vacuum, even pristine samples are exposed to the vacuum for a short period of time, but this time was minimized as much as possible and was on the order of a few minutes. The high-resolution spectra for Pb4f, I3d, C1s, N1s, and F1s (present only in **F-PEA** and **L**) are shown in Figure 2 for 2D perovskites with the five different cations both before and after vacuum exposure. By comparing the spectra, it is evident that exposure to vacuum has a significant effect on the composition of 2D perovskites. In particular, the choice of cation appears to play a critical role in the stability of the perovskite layer since the evolution of the XPS spectra is starkly different for different cations. For example, while **BuA** shows a significant increase in the amount of metallic lead (which appears at a 1.5 eV lower binding energy than the main 2D perovskite Pb peak), no such changes in the spectra of **F-PEA** can be observed. Similarly, we observe that the effect of vacuum on **L** manifests itself as a loss of organic cation, with C, N and F signals significantly decreasing, most likely as a result of the organic cations becoming volatile in vacuum. For other cations, this loss is significantly supressed. We note that for cation **L**, the FWHM of all XPS peaks, especially the N1s peak, is significantly increased after exposure to vacuum. The broadening is most likely the result of subtle changes in the oxidation states of the elements which result in a range of species that are close in binding energy, but are slightly shifted as compared to the pristine peak. The full compositional profiles corresponding to Figure 2 are shown in Figure S1. Additionally, Tables S1 and S2 summarize the atomic percentage values before and after vacuum exposure.

### Dynamics of Degradation

**Figure 3:**
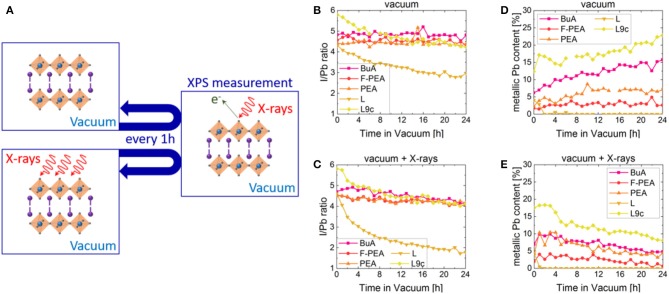
**(A)** Schematic representation of the experimental procedure of vacuum degradation experiments. In each measurement cycle, the XPS measurement took 10 min and then the sample was kept in vacuum or in vacuum with continuous X-ray irradiation for the remaining 50 min. I to Pb ratios of **BuA**, **F-PEA**, **PEA**, **L** and **L9c** over time when exposed to **(B)** vacuum or **(C)** vacuum in combination with X-rays. Evolution of the metallic lead content (with respect to the total amount of Pb) in **BuA**, **F-PEA**, **PEA**, **L**, and **L9c** over time when exposed to **(D)** vacuum or **(E)** vacuum in combination with X-rays.

To investigate in detail the effect of vacuum on the composition of 2D perovskites and in order to evaluate the dynamics of their degradation, XPS measurements were performed once an hour on 2D perovskite samples for a total duration of 24 h. In addition, to identify whether X-ray irradiation also plays a role in the degradation of 2D perovskites, the samples were either solely kept in vacuum in between XPS measurements or were continuously exposed to X-ray irradiation (hν = 1,486.6 eV) throughout the experiment. The experimental procedure is schematically summarized in Figure 3A.

The theoretical composition of 2D perovskites should result in a ratio of I/Pb = 4. Figures 3B,C follow the evolution of this ratio with time in samples exposed to only vacuum and vacuum with X-ray irradiation, respectively. The initial value of I/Pb surpasses the theoretical value of 4 for all samples, with a particularly high value of 5.8 for the **L9c** cation sample. This suggests the presence of excess iodide at the surface of the samples, which has been commonly observed also for 3D perovskites (Sun et al., 2017, 2018; Fassl et al., 2018). Alternatively, the excess could originate from elemental iodine contamination of the parent organic ammonium iodide. In the case of the **BuA**, **F-PEA** and **PEA** samples, the I/Pb ratios range from 4.3 to 4.9 and remain largely constant throughout the measurement. In contrast, **L** and **L9c** exhibit continuously decreasing ratios indicating the loss of volatile iodine. Over the duration of the experiment, the values decrease from 4.4 to 2.8 and from 5.8 to 4.2 for **L** and **L9c**, respectively. When exposing the samples to vacuum and X-rays (Figure 3C), the ratios of **BuA**, **PEA** and **F-PEA** are no longer constant, but decrease to I/Pb≈4 after 24 h. While irradiation does not seem to influence the dynamics of **L9c**, continuously irradiating the **L** sample strongly enhances the decreasing trend observed previously for vacuum only, thereby reducing its I/Pb ratio to a final value of 1.8. These results suggest that X-rays enhance the loss of volatile iodine in vacuum, possibly due to the extra energy provided to the 2D perovskite films. Furthermore, the 2D perovskite containing the **L** cation seems to be the most unstable among the investigated cations, with its final I/Pb≈2 ratio suggesting the formation of lead iodide as a degradation product.

It is important to note that the ratios calculated above include the contributions of all lead and iodine species in order to evaluate the overall loss as a result of vacuum exposure. However, as Figure 2 suggests, the samples exhibit the presence of metallic lead (Pb^0^), which evolves throughout the experiment. Figures 3D,E show the relative contribution of the metallic lead species to the overall Pb content in samples exposed to vacuum and vacuum with X-rays, respectively. We would like to highlight that all the 2D perovskites contain a small amount of metallic Pb even during the first XPS measurement. As mentioned above, XPS studies are performed in vacuum, so samples are exposed to vacuum for several minutes prior to the first XPS measurement during their transfer to the analysis chamber. Thus, it is possible that the observed traces stem from a very fast formation of metallic Pb in vacuum prior to the acquisition of the first measurement. However, it is also possible that traces of metallic Pb are already present in the pristine films, which seems likely for the comparably high starting concentration of metallic Pb in **BuA** and **L9c**.

When exposed to vacuum alone, four of the samples show an increase in the metallic lead content over time. **F-PEA** has the lowest initial percentage of metallic Pb starting at a value of 1.7% and increasing to stable values of around 3% in vacuum. Similarly, **PEA** starts at a value of 2% and stabilizes at ~7% after 12 h in vacuum. **BuA** and **L9c** exhibit continuously increasing percentages of metallic Pb with initial values of 6 and 12% and values just below 16 and 23% after 24 h in vacuum. We propose that upon exposure to vacuum, the 2D perovskites are first degraded into PbI_2_, which further decomposes into metallic lead and volatile ${\text{I}}_{2}^{-}$ ions. It is noteworthy that similar formation of metallic lead was also observed for 3D perovskites when exposed to vacuum conditions (Gunasekaran et al., 2018).

When the samples are irradiated with X-ray in addition to being exposed to vacuum, a different trend is observed. While the initial amounts of metallic Pb stay roughly the same, instead of a continuous increase, we observe an initial short-term increase, followed by a prolonged decrease in the metallic Pb content over time. Consequently, in the course of the 24 h, the metallic Pb content in **F-PEA** is reduced to almost 0%, **BuA** and **PEA** to ~4.5% and **L9c** to ~8% of the overall lead content. The change in dynamics must be attributed to the X-rays. It is likely that while the exposure to vacuum still results in the formation of metallic lead, this species is oxidized due to the presence of a continuous irradiation.

Interestingly, samples with the cation **L** follow a different trend in vacuum as well as in combination with X-ray irradiation. In this case, we observe that the initial metallic Pb content of roughly 4% vanishes within the first 2 h in vacuum and does not reappear during the remaining 22 h of the experiment. More detailed discussion regarding the compositional evolution of samples **L** and **L9c** follows in section Degradation of L and L9c.

To monitor more closely the loss of the organic cations for the various samples, we probed the evolution of the intensity of the C, N, and F elements upon exposure to vacuum (Figure S2). We observe that in **BuA**, **F-PEA**, and **PEA** no significant loss of organic cations takes place. However, in combination with X-ray irradiation, ~5–10% loss is observed in the case of **BuA** and **PEA**. **F-PEA** shows a small decreasing trend in the C, N, and F peak intensities under both conditions. In contrast, **L** and **L9c** show a much stronger decrease in the peak area of the elements related to the organic cation by up to 35 and 25%, respectively, under both conditions. These losses in the contribution of the organic cation to the XPS spectra indicate that the concentration of the organic compound in the 2D perovskite films is reduced, similar to the loss of MAI in 3D perovskites when exposed to vacuum. Interestingly, the loss appears to be smaller for shorter organic cations (**BuA**, **PEA**, **F-PEA**) than for the longer-chained ones (**L**, **L9c**). The exact origin of this effect is topic to ongoing investigation.

### Degradation of L and L9c

**Figure 4:**
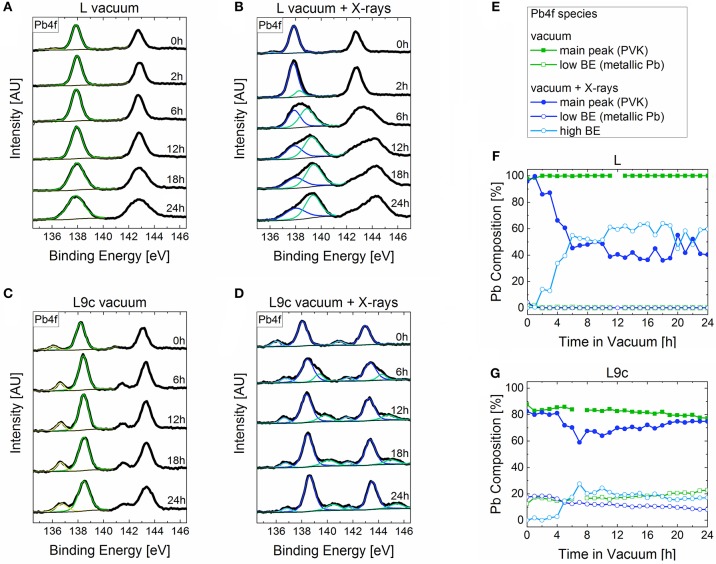
Time evolution of the P4f peaks of **(A) L** in vacuum, **(B) L** in vacuum combined with X-ray exposure, **(C) L9c** in vacuum and **(D) L9c** in vacuum with X-ray exposure with the corresponding time given on the right-hand side of each spectrum. Compositional profiles of the Pb species in **L** and **L9c** when exposed to **(F)** vacuum and **(G)** vacuum combined with X-rays. The legend for panels **(F,G)** is shown in **(E)**.

While the compositional changes in **BuA**, **PEA**, and **F-PEA** are predominantly limited to formation of metallic lead, the changes in the case of cations **L** and **L9c** are more complex. Figure 4 displays the evolution of the Pb4f doublets measured on samples **L** and **L9c** when exposed to either vacuum or vacuum in combination with X-ray irradiation. When exposed to vacuum alone, the Pb4f peak of **L** (Figure 4A) becomes continuously broader with its full width half maximum (FWHM) rising from 0.82 to 1.55 eV after 24 h. This broadening most likely occurs due to subtle changes in the oxidization states but without forming any distinct peak features which makes assigning specific lead species difficult. Likewise, the FWHM of the Pb4f peak of **L9c** in vacuum (Figure 4C) rises from 0.84 to 1.06 eV. With X-rays, the peak broadening is present as well, but additionally a double feature appears at a higher binding energy as compared to the main doublet for both materials (Figures 4B,D). In the case of **L**, the high binding energy species emerges after 2 h in vacuum and makes up 50% of the Pb content after 6 h. In the following hours, its amount fluctuates between 50 and 60%. In the case of **L9c**, this species emerges after 4 h, makes up 30% of the Pb content after 7 h and then continuously deceases to 17% after 24 h. The exact percentages of each Pb species are summarized in Figures 4F,G.

**Figure 5:**
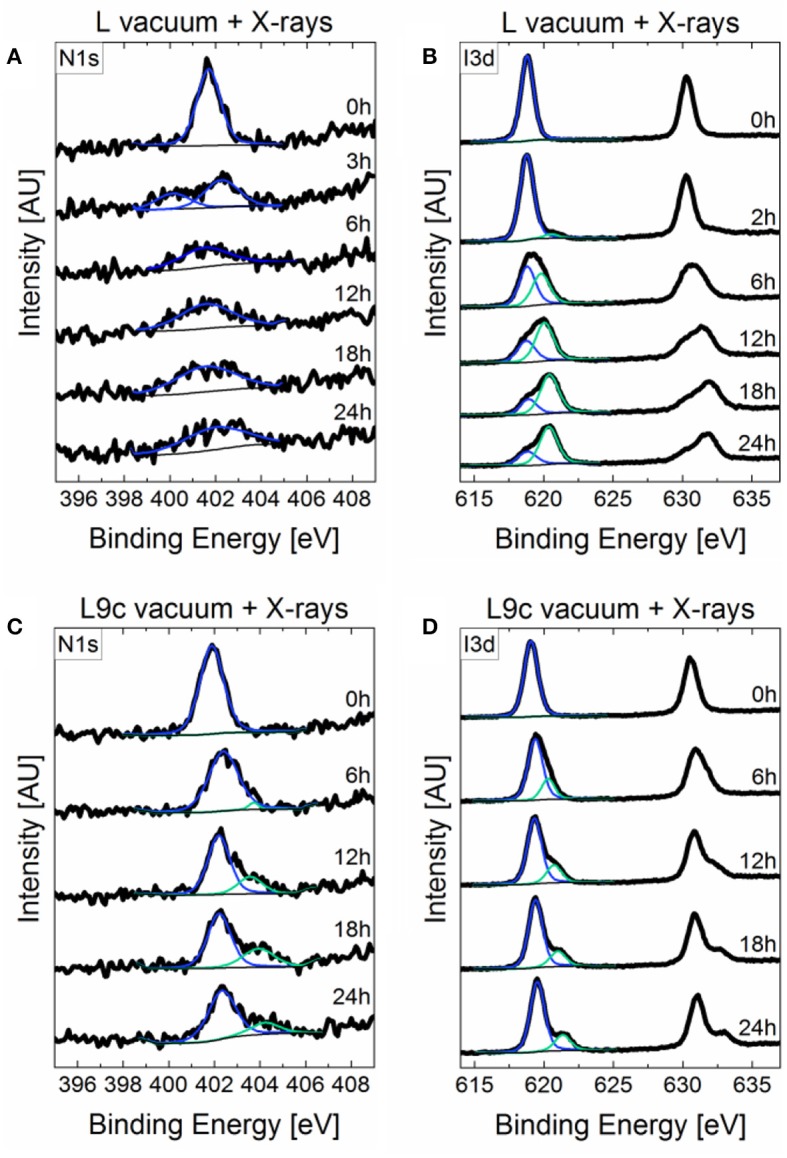
Time evolution of the **(A)** N1s and **(B)** I3d peaks of **L** and the **(C)** N1s, and **(D)** I3d peaks of **L9c** in vacuum combined with X-ray exposure. The corresponding time is given on the right-hand side of each spectrum.

The appearance of the third Pb species must be related to the exposure to X-rays. All other parameters such as vacuum pressure, substrate and XPS measurement time were kept the same. It is possible that the additional energy provided by the X-rays triggered a chemical reaction in the **L** and **L9c** perovskites. The XPS spectra of I3d and N1s support this hypothesis as both these elements show the evolution of new species when exposed to vacuum and X-rays (Figure 5). Alternatively, the Pb peak could be made up of several oxidation states up to Pb^4+^ or could form due to surface charging. The exact identification of this species is a topic of future work.

### Changes in Layer Microstructure and Bulk Properties

**Figure 6:**
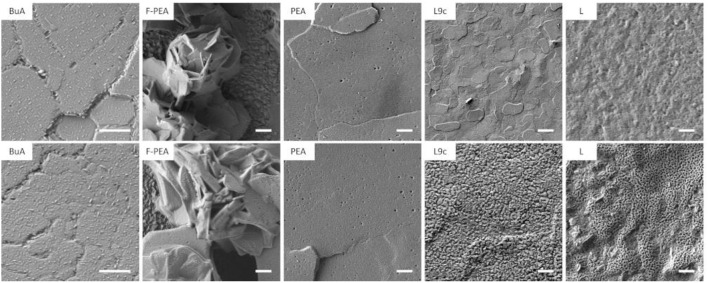
Scanning electron microscope images of the pristine film **(top)** and films after several hours of vacuum exposure **(bottom)**. Scale bar 1 μm.

In addition to a strong influence on the composition of 2D perovskites, exposure to vacuum may strongly affect their microstructure. To evaluate this effect on the sample surface morphology, samples were imaged using scanning-electron microscopy (SEM) both before and after exposure to vacuum (Figure 6). The samples containing cations **L9c** and **L** show the strongest changes in surface topography upon exposure to vacuum. In case of **L9c** the relatively smooth surface of platelets of ca. 1 μm size becomes more irregular and rougher. The degraded film exhibits a grainy surface texture of agglomerates of only 10–100 nm in size. Larger platelets of 2D-perovskites as in the pristine film are no longer observed. In the case of cation **L** the overall porosity and roughness of the film is dramatically increased and the exposure to vacuum leaves a “sponge-like” scaffolding behind. These obvious changes in the surface morphology are in line with the strong compositional changes of the 2D perovskite film in case of cations **L** and **L9c**. Pristine films with cations **PEA**, **F-PEA**, and **BuA** consist of crystalline material with significantly larger domain sizes compared to films of **L** and **L9c**. Upon exposure to vacuum no apparent morphological change can be identified in these three films. The crystalline domains seem to remain mainly intact and the surface structure remains largely unaffected. Films of **F-PEA** perovskite do not cover the substrate completely (in agreement with observed XPS signal from the FTO substrate, Figure S1) and form agglomerates of crystalline perovskite platelets. **BuA** and **PEA** form homogenous and compact films. For all three cations the perovskite crystallites feature a common appearance: all platelets exhibit small holes and a texture on the platelet surface. In the case of **PEA** this texture consists of small crystallites, dozens of nm long, growing only along two perpendicular directions on top of each grain. Exposure to vacuum might affect the described surface texture, however changes are too subtle to be reliably identified in this experiment and might require longer exposure times. Furthermore, we cannot fully exclude that even the transfer of the samples in the vacuum of the SEM and the exposure to the electron beam already caused changes even on the pristine samples. To exclude effects of vacuum and electron beam exposure, we imaged the film surfaces also using atomic force microscopy (AFM) in ambient conditions before and after exposure to vacuum (Figure S3). The AFM images show generally similar changes in the surface structure of the layers, confirming that they occurred during the exposure to vacuum, and are not the result of SEM imaging experiments.

While XPS and SEM provide valuable information about the degradation of the surface of 2D perovskites upon exposure to vacuum, it is important to investigate its effect also on the bulk properties of the layers. For this purpose, we collected X-ray diffraction (XRD) and UV-VIS spectra prior and after vacuum exposure. As can be seen in Figure S4, the XRD spectra exhibit the characteristic features of 2D perovskites confirming their crystalline structure. Furthermore, the same diffraction patterns are observed after vacuum exposure with no changes to the position or width of the XRD reflections. Furthermore, no new reflections appear in the spectra upon degradation, suggesting that the degraded species are predominantly non-crystalline. Figure S5 shows the UV-VIS spectra collected prior and after degradation. The absorption spectra of **BuA**, **F-PEA**, and **PEA** remain largely unaffected by vacuum degradation, with only a slight blue-shift by ~3–5 nm and a broadening in the case of **BuA** and **F-PEA**. We note that the large roughness of sample **F-PEA** results in significant scattering, which manifests itself as an increased absorbance over the entire spectrum. However, in the case of **L** and **L9c** the degradation induces a stronger loss in absorbance which in combination with the observations from SEM and AFM suggests that the degradation is not limited to the surface but affects also the bulk of the sample. This could originate from the porous structure observed for the degraded samples in SEM, which triggers the degradation of deeper layers. Therefore, we conclude that vacuum degradation under the conditions tested in this study is mostly limited to the surface for cations **BuA**, **F-PEA**, and **PEA**, but has a more pronounced effect on the bulk properties of the 2D perovskites based on **L** and **L9c** cations.

## Conclusions

We demonstrate that vacuum exposure induces the formation of a metallic lead species which, in the cases of the organic cations **L** and **L9c**, is accompanied by a loss of the organic cation from the perovskite. Investigating the dynamics of this reaction revealed that the metallic lead content increases while the organic content decreases over time in vacuum with the exact slope being dependent on the choice of organic cation. When exposed to vacuum with constant X-ray illumination, the dynamics change, and we observe a decrease in the metallic lead content. Additionally, X-rays may cause or enhance the loss of volatile iodine depending on the cation. In the case of L and L9c, X-rays may trigger a chemical reaction which leads to the formation of a third lead species at a higher binding energy indicating severe degradation. In agreement with the XPS results, we see that the microstructure of L and L9c drastically changes upon vacuum exposure while **BuA**, **F-PEA**, and **PEA** remain largely unaffected.

Our study emphasizes the importance of selecting suitable organic cations for 2D perovskites to prevent degradation. The exact chemistry behind such degradation should be identified and investigated in detail in order to develop suitable mitigation strategies. Until then, these findings should be taken into considerations when fabricating and measuring 2D perovskite films on their own or as capping layers on top of 3D perovskites. Many commonly used fabrication routines include vacuum deposition of, for example, the perovskites themselves as well as hole-transport layers, passivating layer and contacts on top of the perovskites. It is possible that exposure to vacuum during these processes commences the degradation of the 2D perovskites or hinders the formation of a proper film. Furthermore, degradation may also happen during spectroscopic or microscopic measurements which take place in vacuum, which would gradually alter the results as the measurement is carried out.

## Experimental

### 2D Perovskite Sample Fabrication

First of all, PbI_2_ was dissolved in dimethyl sulfoxide (DMSO) at a concentration of 1.2 M at 60°C. After the solution had cooled down, 2.4 M of the organic cation (**BuA**, **F-PEA**, **PEA**, or **L9c**) was added to the solution. In the case of (L)_2_PbI_4_, the concentration of the precursors was reduced to get the optimal thickness. In particular, 1 M of fluorinated cation **L** was added to 0.5 M of PbI_2_ in DMSO (García-Benito et al., 2020). Then, the perovskite solutions were deposited onto the FTO substrate via a consecutive two-step spin-coating process at 1,000 rpm for 10 s and 5,000 rpm for 30 s. During the second step, 100 μL of chlorobenzene was deposited as an antisolvent. No antisolvent was used for the formation (L9c)_2_PbI_4_ perovskite. Lastly, the resulting films were annealed at 100 °C for 10 min. The resulting film thickness is ~500 nm.

### X-ray Photoemission Spectroscopy (XPS)

The samples were transferred into an ultrahigh vacuum (UHV) chamber of the PES system (Thermo Scientific ESCALAB 250Xi) for measurements. The chamber pressure was 2 × 10^−9^ mbar. XPS measurements were performed using an XR6 monochromated Al Kα source (hν = 1,486.6 eV) and a pass energy of 20 eV. The sample surface was connected to the sample holder with a copper clip to prevent charging. Vacuum-induced degradation was monitored by performing a series of XPS measurements every 60 min (60 min = one measurement cycle) and analyzing the compositional changes. Two spots were measured on each sample. The X-ray spot size was 650 μm and the distance between the two measurement spots was 5 mm to prevent illumination through scattered X-rays. The spot “vacuum” was exposed to X-rays for only 10 min during each measurement cycle to collect the XPS spectra. The spot “vacuum + X-rays” was exposed to X-rays for a total of 50 min during each measurement cycle including the 10 min needed to collect the XPS spectra.

### Scanning Electron Microscopy (SEM)

The samples were imaged prior and post degradation in UHV for 24 h. SEM imaging was performed using a JSM-7610F FEG-SEM (Jeol). Samples were mounted on standard SEM holders using conductive Ag paste on the exposed FTO corner to avoid sample charging. The images were recorded using the secondary electron detector (LEI) at an acceleration voltage of 1.5 kV and a chamber pressure <10^−6^ mbar.

### Atomic Force Microscopy (AFM)

AFM images (5 × 5 μm^2^) were taken in tapping mode in ambient atmosphere on an AIST-NT Combiscope1000 using NANOSENSORS™ PPP-NCHR probes. Pristine samples were used after fabrication of the perovskite layers. Degraded samples were exposed to vacuum (2 × 10^−7^ mbar) for 36 h and imaged directly afterwards. Samples with cation **F-PEA** could not be imaged with AFM due their high surface roughness.

### UV-VIS Spectroscopy

The samples were measured prior and post degradation in vacuum (2 × 10^−7^ mbar) for 36 h with a Shimadzu UV-3100 UV-VIS-NIR recording spectrometer. An empty FTO substrate was used for the baseline measurement.

### X-ray Diffraction (XRD)

X-ray diffraction (XRD) measurements were conducted in ambient on a Bruker Advance D8 diffractometer equipped with a 1.6 kW Cu-Anode (λ = 1.54060 Å) and a LYNXEYE_XE_T detector operated in 0D-Mode. The scans (from 2θ = 2°-60°, step size 0.01°, 0.2 s/step) were measured in Standard Bragg-Brentano Geometry (goniometer radius 420 mm) with IS = 0.2 mm and RS = 0.2 mm and a 25 mm beam mask. The measured data was background (subtracted FTO/glass-measurement) corrected and contribution of Kα2 was stripped using the Diffrac.Eva V4.3 software. The films were scanned prior and post degradation in vacuum (2 × 10^−7^ mbar) for 36 h.

## Data Availability Statement

All datasets generated for this study are included in the article/Supplementary Files.

## Author Contributions

YH performed the degradation experiments, XPS measurements and analysis, and UV-VIS measurements. SO synthesized the L9c cation. IG-B fabricated the samples. FP performed the SEM and AFM imaging and XRD measurements. GG and YV guided and supervised the project. YH and YV wrote the manuscript, which has been edited by all co-authors.

### Conflict of Interest

The authors declare that the research was conducted in the absence of any commercial or financial relationships that could be construed as a potential conflict of interest.
