# Supplementary material for: Vacuum-Induced Degradation of 2D Perovskites
Source: Front Chem. 2020 Feb 13;8:66. doi: 10.3389/fchem.2020.00066 (PMC7031494; doi:10.3389/fchem.2020.00066)
Supplement: Supplementary file 1 [file Data_Sheet_1.PDF]

Supplementary Information

Vacuum-induced degradation of 2D perovskites

Yvonne J. Hofstetter,[1,2] Inés García-Benito,[3] Fabian Paulus,[1,2] Simonetta Orlandi[4], Giulia Grancini,[3,5] and Yana Vaynzof[1,2*]

[1] Kirchhoff Institute for Physics and the Centre for Advanced Materials, Im Neuenheimer Feld 227, Heidelberg University, 69120 Heidelberg, Germany

[2] Integrated Centre for Applied Physics and Photonic Materials and Centre for Advancing Electronics Dresden (cfaed), Technical University of Dresden, Nöthnitzer Straße 61, 01187 Dresden, Germany

[3] Group for Molecular Engineering of Functional Materials, Institute of Chemical Sciences and Engineering, EPFL Valais Wallis, Rue de l'Industrie 17, CP 440, CH-1951 Sion, Switzerland

[4] CNR - Istituto di Scienze e Tecnologie Chimiche "G. Natta" (CNR-SCITEC), Via Golgi 19, 20133 Milano, Italy

[5] Department of Chemistry, University of Pavia, Via Torquato Taramelli 14, 27100 Pavia, Italy

*** Correspondence:**
Corresponding Author: yana.vaynzof@tu-dresden.de

Table S1: Atomic percentages of the 2D perovskites with organic cations **BuA**, **F-PEA**, **PEA**, **L** and **L9c** at 0 h and 24 h in vacuum. Values correspond to the PB4f, C1s, N1s, I3d and F1s spectra shown in Figure 1 in the main text as well as the complete compositional profiles in Figure S1. Values are rounded to 2 decimals.

		**Pb4f [at.%]**	**C1s [at.%]**	**N1s [at.%]**	**I3d [at.%]**	**F1s [at.%]**	**Sn3d [at.%]**	**O1s [at.%]**
**BuA**	**0 h**	5.32	55.02	13.96	25.69	-	-	-
	**24 h**	5.16	55.10	14.95	24.80	-	-	-
**F-PEA**	**0 h**	2.81	55.16	6.50	12.82	8.09	3.69	10.93
	**24 h**	3.06	54.50	6.35	13.25	8.38	3.83	10.62
**PEA**	**0 h**	4.49	66.60	9.07	19.84	-	-	-
	**24 h**	4.49	66.87	8.90	19.74	-	-	-
**L**	**0 h**	1.79	35.08	3.16	7.69	52.28	-	-
	**24 h**	3.61	33.45	3.59	10.77	48.58	-	-
**L9c**	**0 h**	1.78	72.58	9.15	10.28	-	-	6.22
	**24 h**	3.02	70.04	8.50	12.77	-	-	5.67

Table S2: Atomic percentages of the 2D perovskites with organic cations **BuA**, **F-PEA**, **PEA**, **L** and **L9c** at 0 h and 24 h in vacuum with X-ray illumination. Values are rounded to 2 decimals.

		**Pb4f [at.%]**	**C1s [at.%]**	**N1s [at.%]**	**I3d [at.%]**	**F1s [at.%]**	**Sn3d [at.%]**	**O1s [at.%]**
**BuA**	**0 h**	5.31	56.23	13.46	25.00	-	-	-
	**24 h**	6.68	53.56	12.14	27.62	-	-	-
**F-PEA**	**0 h**	2.72	55.20	6.81	12.38	7.87	3.74	11.26
	**24 h**	3.58	52.63	5.97	14.55	7.49	4.26	11.52
**PEA**	**0 h**	4.35	66.71	9.39	19.55	-	-	-
	**24 h**	4.87	66.18	8.75	20.20	-	-	-
**L**	**0 h**	1.55	33.59	3.33	7.14	54.38	-	-
	**24 h**	4.58	36.34	3.32	8.19	47.57	-	-
**L9c**	**0 h**	1.79	74.08	8.68	10.43	-	-	5.02
	**24 h**	3.35	71.28	7.64	13.78	-	-	3.96

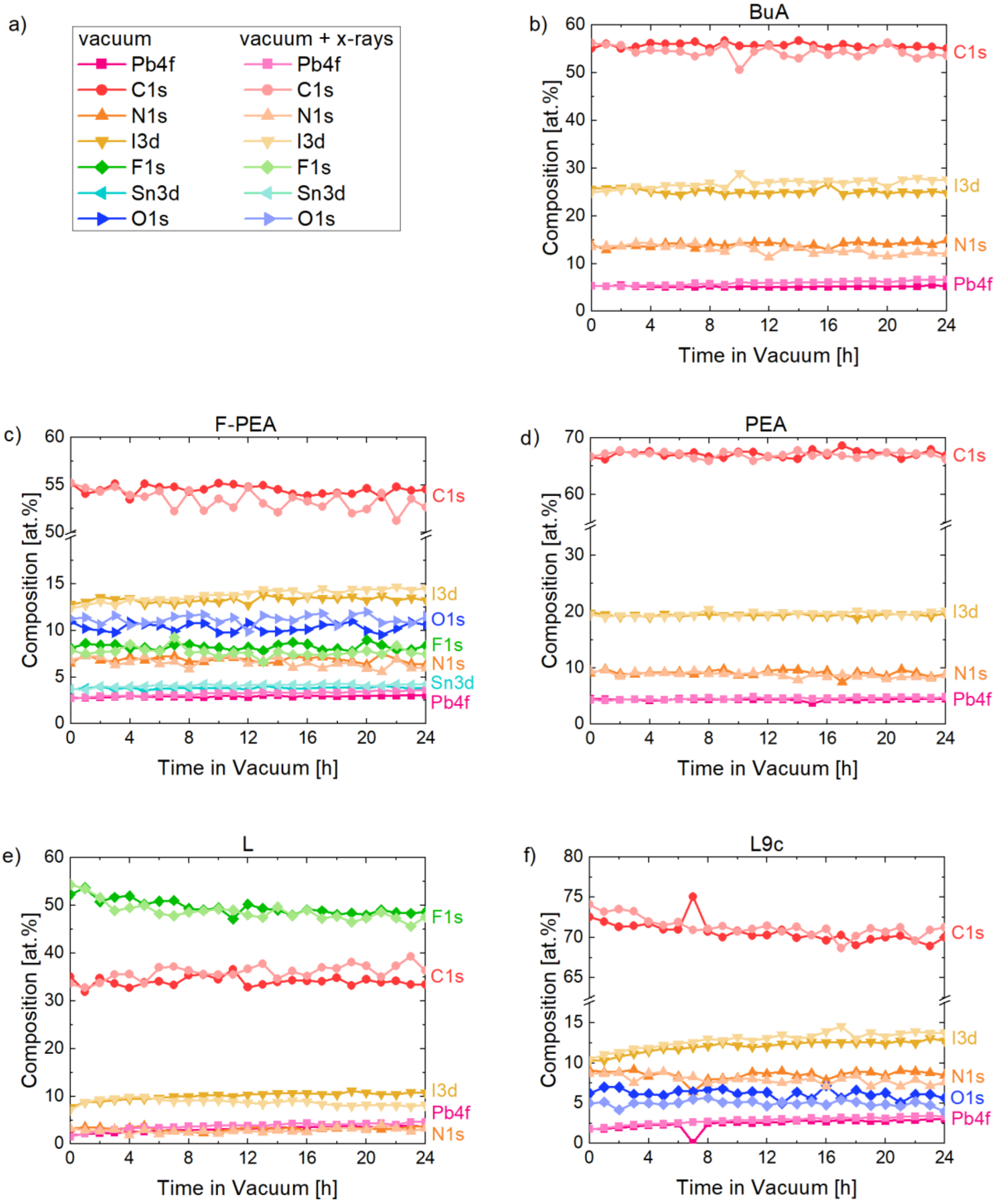

Figure S1: Compositional profiles of b) **BuA**, c) **F-PEA**, d) **PEA**, e) **L** and f) **L9c** comparing the effect of exposure to vacuum and exposure to vacuum combined with X-rays. The legend for all panels is shown in a).

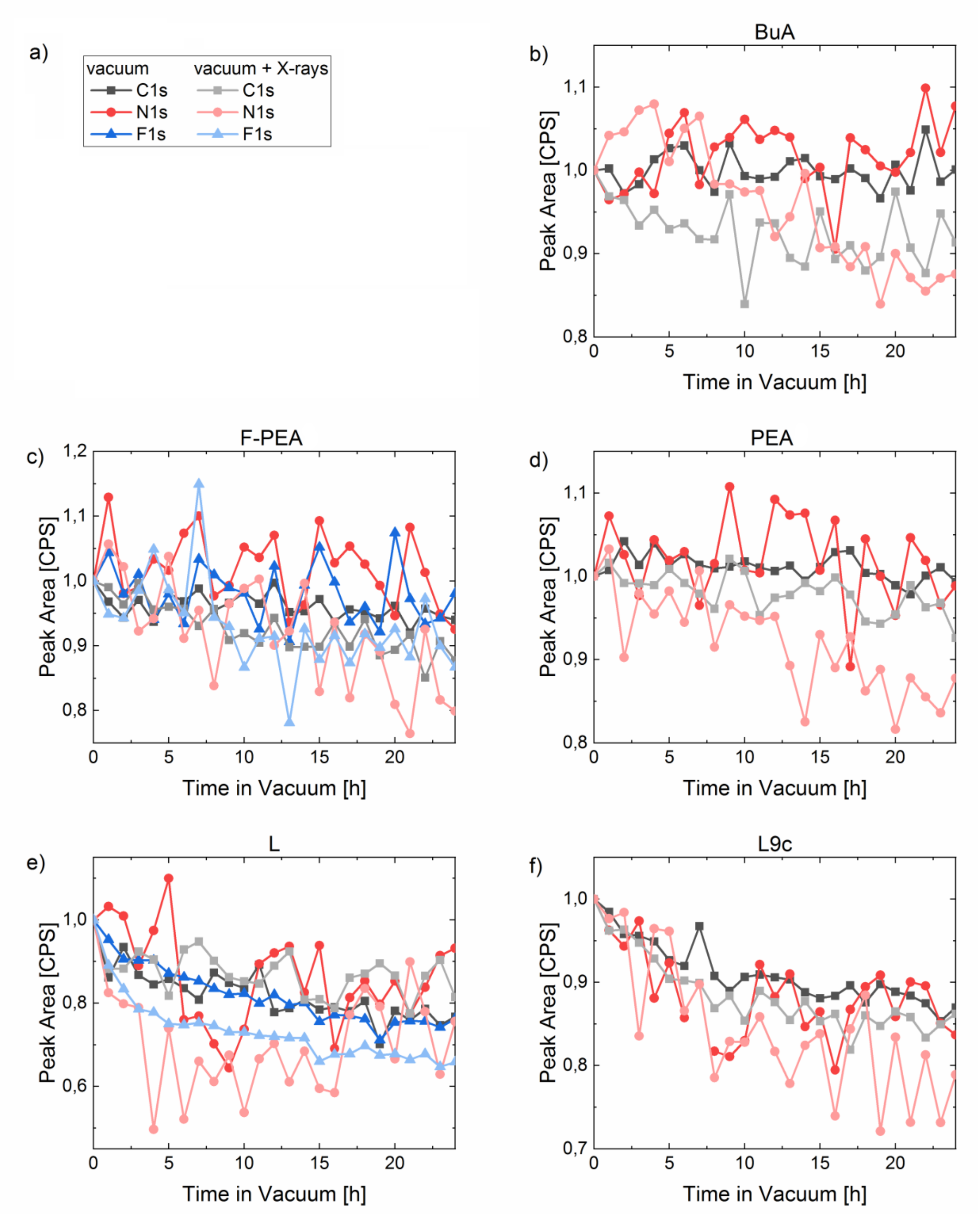

Figure S2: Normalized peak area profiles of the organic components in b) **BuA**, c) **F-PEA**, d) **PEA**, e) **L** and f) **L9c** comparing the effect of exposure to vacuum and exposure to vacuum combined with X-rays. The legend is shown in panel a).

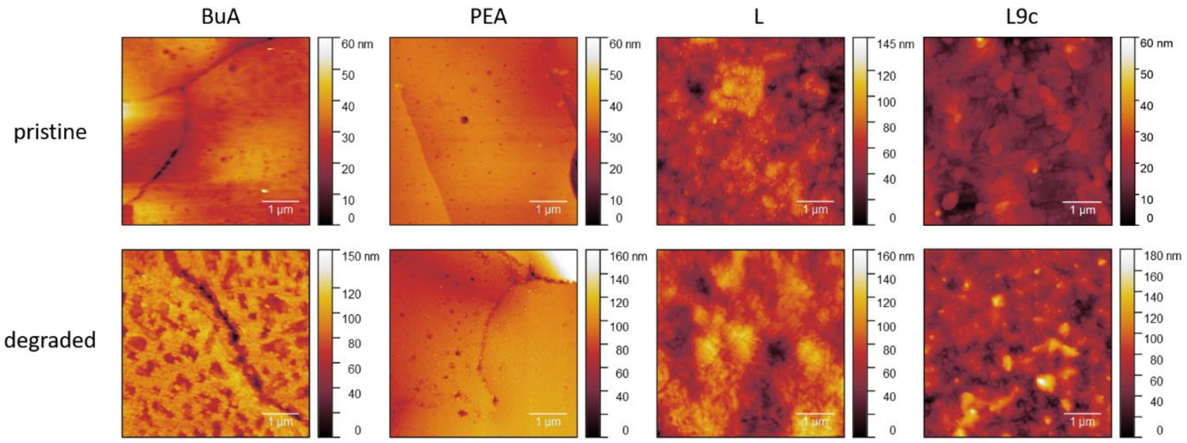

Figure S3: AFM measurements of the pristine and vacuum degraded 2D perovskites. **F-PEA** could not be measured with AFM because of its high surface roughness.

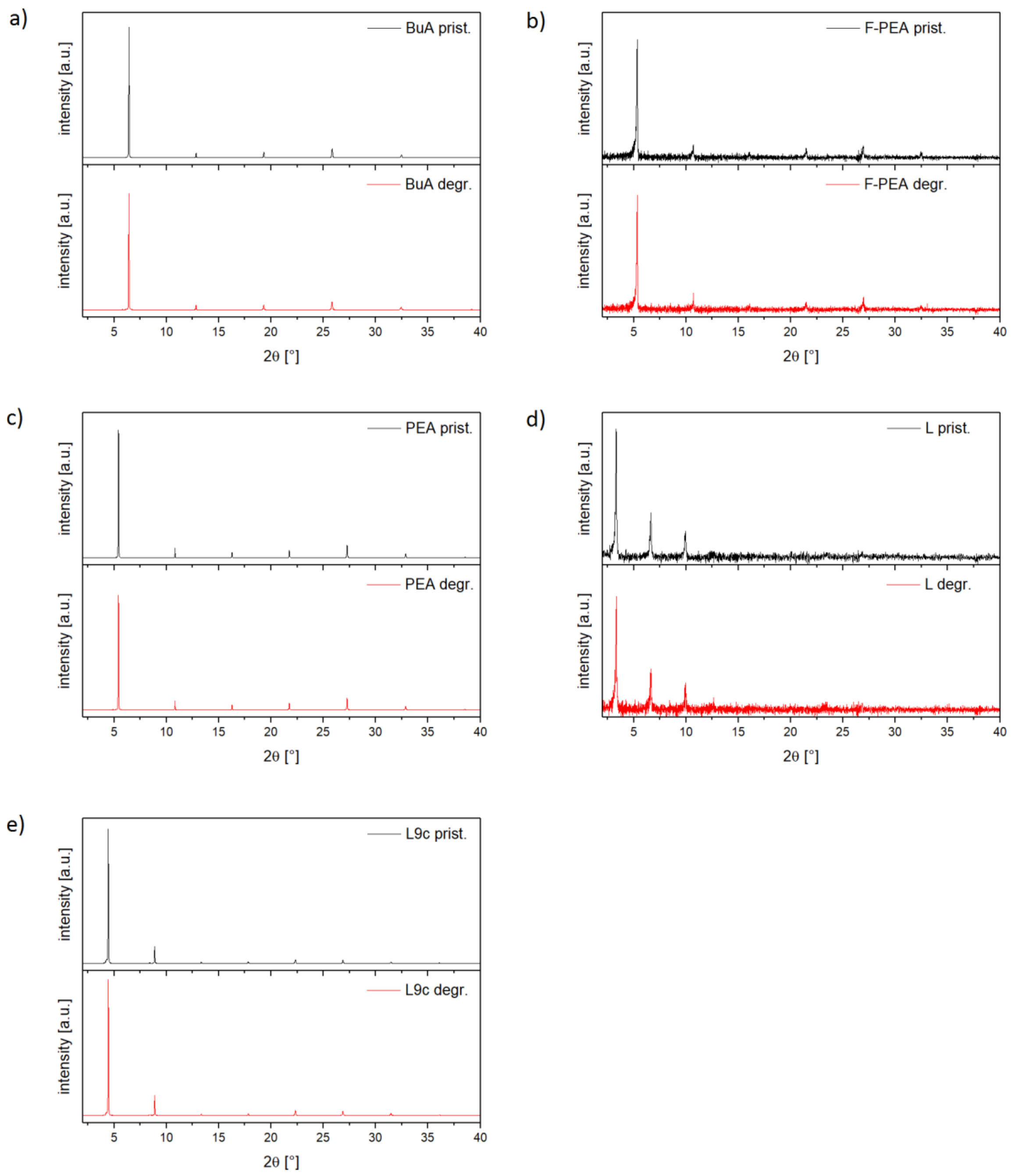

Figure S4: XRD measurements of the pristine and vacuum degraded 2D perovskites a) **BuA**, b) **F-PEA**, c) **PEA**, d) **L** and e) **L9**.

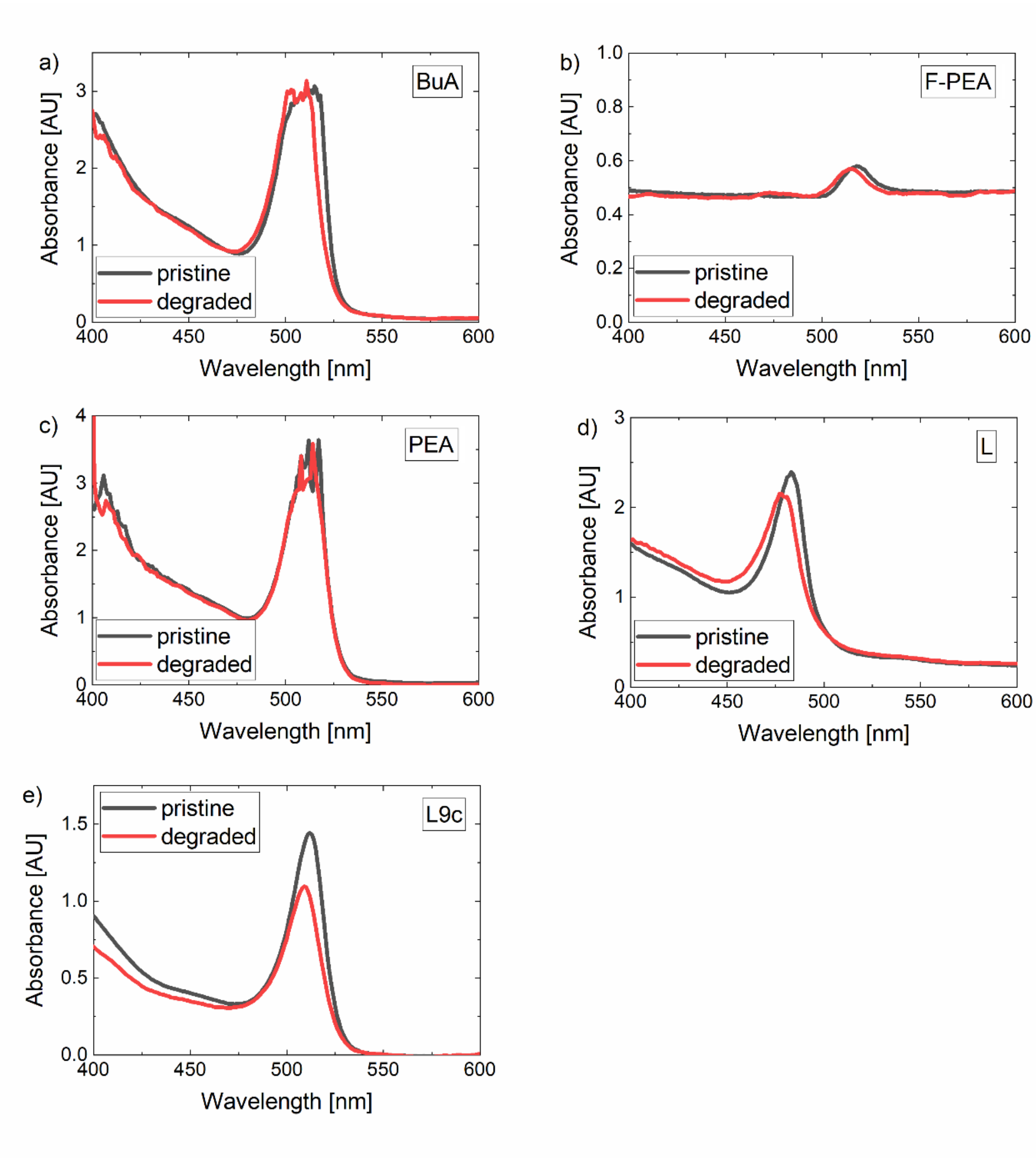

Figure S5: UV-VIS measurements of the pristine and vacuum degraded 2D perovskites a) **BuA**, b) **F-PEA**, c) **PEA**, d) **L** and e) **L9c**.
